# Social support as a correlate of depression among people living with HIV and AIDS in Nigeria

**DOI:** 10.4314/ahs.v21i3.9

**Published:** 2021-09

**Authors:** Dorothy Ebere Adimora, Francisca Ngozi Ogba, Monica Obiageli Omeje, Fidelis Eze Amaeze, Friday Mamudu Adene

**Affiliations:** 1 Department of Educational Foundations, Faculty of Education, University of Nigeria, Nsukka; 2 Department of Educational Foundations, Alex-Ekwueme, Federal University, Ndufu Alike, Ebonyi State, Nigeria; 3 Department of Arts Education, Faculty of Education, University of Nigeria, Nsukka

**Keywords:** Depression, instrumental support, emotional support, HIV, AIDS

## Abstract

**Background:**

Depression is a highly prevalent mental disorder among PLHIV, whilst social support is important in disease prevention, health promotion, therapeutic measure especially for PLHIV.

**Objectives:**

To ascertain the different types and sources of social support and their association with depression among PLHIV in Nigeria.

**Materials and Methods:**

The study was a correlation with 2515 PLHIV in three teaching hospitals in South-Eastern Nigeria. Data were collected between January to June, 2019 through interviews, using socio-demographic and Clinical Form and a Social Support Scale for PLHIV. SPSS-20 used for data analysis.

**Results:**

It was shown that average scores of instrumental and emotional social supports (IESS) were satisfactory and not influenced by sex (p = 0.894; p = 0.496), education (p = 0.805; p = 0.182), marital status (p = 0.076; p = 0.446) and length of antiretroviral therapy (p = 0.510; p = 0.136). People diagnosed for less than three years had more instrumental support (p = 0.05) than those diagnosed over three years. The regression score also revealed a high predictive power of IESS on depression of PLHIV.

**Conclusion:**

PLHIV have satisfactory social support, especially from family not residing in the same household and emotional social support from friends. Analyses identified knowledge gaps in the community regarding the social support received by PLHIV and their depression symptoms.

## Introduction

HIV/AIDS is a chronic infectious disease and firstleading cause of mortality and morbidity worldwide from infectious diseases^1^. An estimated 2.1 million adolescents (aged 10–19 years) are living with HIV, the vast majority in sub-Saharan Africa^1^. Nearly half of new HIV infections occur in young people (aged 15–24 years)^1^. Similarly, an estimated 5 million young people aged 15–24 years are living with HIV, vast majority in sub-Saharan Africa^1^.

Nigeria has the second largest HIV epidemic in the world^2^. Although HIV prevalence among adults is much less (2.8%) than other sub-Saharan African countries such as South Africa (18.8%) and Zambia (11.5%), the size of Nigeria's population means that 3.1 million people were living with HIV in 2017^3^. However, a recently published Nigeria HIV/AIDS Indicator and Impact Survey (NAIIS), one of the largest population-based HIV/AIDS household surveys ever conducted, found the prevalence to be just 1.4%^4^. The apparent decline has been attributed to better surveillance^4^. People living with HIV/AIDS (PLHIV) are particularly vulnerable for a variety of biological, behavioural, social and structural reasons, depression and providing holistic care to this group presents many unique challenges^5^.

According to DSM-5 depression is a common mental disorder characterized by depressed mood, loss of interest (pleasure), decreased energy, feeling of guilt, or low self-worth, disturbed sleep or appetite, and poor concentration^6^;^5^. Depressive disorders were the third leading cause of global burden of disease in 2004 and will be the first by 2030. Globally, depression is the major cause of illness and disability among youth^7^.

In the recent time, researchers have demonstrated high interest in the field of HIV/AIDS, and publication of several studies highlighted the high levels of depression in PLHIV, as the case may be, there continues to be a dearth of evidence related to PLHIV and very little data examining the associated risk factors and correlates of depression in the HIV-infected population, especially in the African socio-cultural context. Studies in the West have pointed to a multifactorial aetiology of depression in HIV/AIDS that includes psychological, social, and biological factors. The few studies that have been conducted in sub-Saharan Africa suggest the following risk factors for depression in PLHIV: female gender, older age, unemployment, negative life events, childhood trauma, impaired function, poor quality of life, low CD4 counts and poor social support^8^;^9^. Social support means having friends and other people, including family, to turn to in times of need or crisis for a broader focus and positive self-image. Social support enhances quality of life and provides a buffer against adverse life events10. Social support is a dimension of the Social Determinants of Health (SDH), defined as a set of social, economic, cultural, psychological, ethnic/racial and behavioral factors that influence health. The main SDH analysis model is from Dahlgren and Whitehead, which is arranged in five concentric layers, ranging from individual determinants (inherent to the person and not modifiable) to macrodeterminants (made up of economic, cultural and environmental conditions)^11^. The two types of social support -Emotional-affective and instrumental-operational. Emotional (sometimes called non-tangible) support refers to the actions people take to make someone else feel cared for. Instrumental or operational support refers to the physical, such as money and housekeeping^10^. The emotional-affective support includes activities related to attention, presence and listening, so that the person feels cared for or appreciated; whereas the instrumental or operational support regards household chores or practical aspects of the treatment itself, like accompanying the person in a medical visit, fetching medicines in the health unit, taking care of the children during medical visits, amongst other activities^12^. Research reveals that social support is an important variable in the prevention of diseases, promotion of health, therapeutic compliance and in the process of recovery from illness^13^. It has a protective effect during crisis, such as mourning, retirement, unemployment, illness recovery, and hospitalization, as well as the HIV infection12. HIV infection could lead to depression especially among young people. It has also been correlated with high risk behaviours such as earlier sexual debut, substance abuse, more frequent sexual partners, and unplanned pregnancy^13^;^14^. Living with HIV and AIDS can as well increase the risk of mental illness such as depression. Even though neuropsychiatric comorbidities (including depression) are highly prevalent among HIV-positive adolescents worldwide, there are no studies which show prevalence of depression among PLHIV-positive in Nigeria.

Depression is a result of mood disorder with several features such as sadness, pessimism, decrease in activity, fatigue, weight-loss, sleeplessness, or too much sleep and feeling of unnecessary guilt. Severe depression could make individuals commit suicide. According to^15^ depression is caused by disturbances in cognitive, interpersonal, neuro-chemical and environmental functioning as well as deficits in critical emotion regulation skills. Therefore, determining prevalence of depressive symptoms and associated factors among HIV-positive individuals is important for early intervention and further decreases the burden of depression and contributes to have a plane for improving patients' quality of life. The moderating factors for depression in PLWHA were age, gender, primary care giver type, maternal death, change in care giver type, death in the family 17;18;19 failing in the school term, medication adherence, HIV related stigma, and poor social support which were the major moderating factors for depression among HIV-positive youth^14^;^20^;^21^.

This study, therefore seeks to investigate the relationship between social support and depression among PLHIV in Nigeria, determine the demographic factors that predict depression and the predictive power of instrumental and emotional social supports on depression of PLHIV

## Methods and materials

### Study Settings and Populations

An institution based cross sectional study was conducted in University of Nigeria Teaching Hospital Enugu - 708; Federal Medical Centre Umuahia in South-Eastern Nigeria - 986; and Federal Teaching hospital, Abakaliki, Ebonyi State - 821, totaling 2515 in the three teaching hospitals in South-Eastern Nigeria.

### Sampling Technique

The participants were 1242 (434 male and 808 female) people living with HIV and AIDS drawn through purposive sampling of HIV patients who have consented for the study. Only those who gave their consent were used for the study therefore, there was no need for application of other sampling techniques.

### Reliability

Reliability of the interview questions and the questionnaire item statements was ascertained in the University of Port-Harcourt Teaching Hospital (UPTH) before the main study since it is not in the area of this study. Data collected were analyzed using Cronbach alpha and a coefficient of consistency of 0.91 was established for BDI-11, whilst 0.87 was for Social Support Inventory (SSI). A test-retest reliability to determine the stability of the instrument was conducted overtime.

### Inclusion and Exclusion Criteria

Participation was voluntary, informed verbal consent was obtained from all patients before their inclusion in the study. The selection was made from the participants who were visiting the hospital at the time of this research and who adhere to medications and medical appointments.

### Measures

Beck Depression Inventory-II (BDI-II) was used to assess the presence and severity of depressive symptoms. The tool consists of 21 items and patients with a score greater than or equal to 2122 were taken as having depressive symptoms. The BDI-II is one of the most commonly used tools in research. The BDI-II has been used in more than 2,000 studies and translated into many languages^22^, and it is the most commonly used measure of depression in studies involving subjects with HIV. The current form of the tool is the BDIII, 1 a 21-item, self-report instrument for measuring the severity of depression in adults and adolescents age 13 and older^23^. 22 listed as advantages of the BDI-II its high internal consistency, high content validity, its specificity in differentiating between depressed and nondepressed subjects, its sensitivity to change, and its international popularity. The BDI-II was developed for the assessment of symptoms corresponding to criteria for diagnosing depressive disorders listed in the Diagnostic and Statistical Manual of Mental Disorders, 4^th^ edition (DSM-IV)^24^. For the diagnosis to be made, five (or more) of the symptoms listed in Table 1 have to be present during the same 2-week period and to represent a change from previous functioing; at least one of the symptoms has to be either depressed mood or loss of interest or pleasure.

**Table 1:** Sociodemographic categorization of PLWHA

Sociodemographic Variables	No	Percentage %
***Gender***		
*Male*	534	43.00
*Female*	708	57.00
***Age***		
*Below 35 years*	686	55.23
*Above 35 years*	556	44.77
***Number of HIV-Related Symptoms*** ***Duration from HIV diagnosis***		
*Below 1 year*	13	.01
*1–2 years*	120	9.66
*3–4 years*	252	20.29
*Above 5 years*	857	69.00
***Duration from ART initiation***		
*Below 1 year*	138	11.11
*1–2 years*	199	16.02
*3–4 years*	416	33.49
*Above 5 years*	489	39.37
***Marital Status***		
*Married*	56	4.51
*Unmarried and without a partner*	1004	80.84
*Unmarried but with a partner*	92	7.41
*Others (divorced, widow, etc)*	90	7.25
***Educational attainment of household members***		
*Low*	625	50.32
*Middle*	402	32.37
*High*	215	17.31
***Employment***		
*Employed*	504	40.58
*Unemployed*	526	42.35
*Retired*	212	17.07
***Individual Income***		
*Low*	534	44.61
*Middle*	482	38.81
*High*	206	16.59
***Health Insurance***		
*No*	719	57.89
*Yes*	523	42.11
***Disclosure Status***		
*No*	89	27.17
*Yes*	1153	72.83

A reliability coefficient of 0.88 was reported for the scale and found to be a valid measuring device when compared with other instruments^25^. For each of the 21 items, the participant has three possible answers; 0 indicating an absence of symptoms, 1 indicating mild symptoms, and 2 indicating definite symptoms. The total score ranged from 0 to 54, with higher scores representing more severe depressive symptomatology. Construct, content and face validity of the instruments were ensured. Participants were classified according to cut-offs proposed by^25^, which minimise the risk of false positives, whereby a BDI-II score of 0 indicates no symptoms, scores 1–19 indicate ‘mild to moderate’ depressive symptoms and scores equal to or above 20 indicate ‘definite caseness’^25^;^26^. This classification was applied since there was no specific cut-off point for BDI-II based on studies carried out on Nigerian adolescents. Researchers argued that a lower cut-off point is only usually suggested for populations where high rates of depression are expected^27^.

### Social support

Social support included measures of provision and receipt. The measure of the amount and scope of help that participants could receive or provide to others in their family, friends, and their community as gifts, money, supplies, etc. in the last 12 months. Providing (all) ranged from 0 to 18 and larger values indicate greater levels of providing help or support. Social support is a measure of the amount and scope of received help or support that participants received from others (family friends, community and government). The measure of receiving (all) ranged from 0 to 15 and larger values indicate that participants received greater levels of help or support. Types of social support were evaluated using the Social Support Inventory^28^. This is a self-administered scale developed to measure perceived adequacy of social supports and availability of types of social support. Social Support Inventory (SSI) consists of 20 items representing two types of social support; emotional/instrumental support. The score for each item ranges from (rarely or none of the time) (all of the time). SSI has been translated into various languages and adapted to different cultures and contexts. Summed scores in each type of social support were used as continuous variables and categorical variables (quartiles)n the descriptive analyses, and as continuous variables in other analyses.

Information on sources of each type of social support in SSI was added to the questionnaire. Social support consists of material and psychological resources to which people have access through social networks^29^. It is subdivided according to two factors: emotional social support which involves being listened to, attention, information, esteem, company and emotional support; and instrumental which represents support in operational matters related to treatment or health care and in practical day-to-day activities, in addition to material and/or financial help^30^.

Considering the predictive nature of social support on the depressive symptoms of PLWHA, it becomes imperative to evaluate social support based on a model that takes into consideration the multiple dimensions of depression and the remediation process, in order to provide input for health professionals to identify and coordinate support networks that facilitate health care. In this context, the present study sought to analyze the social support of people living with HIV/AIDS from the perspective of the Social Determinants of Health Model (SDHM).

### Demographics and HIV-related factors

The following data on demographic and HIV-related factors were collected: gender, age, Number of HIV-Related symptoms: duration from HIV diagnosis, duration from ART initiation, marital status, Educational attainment of household members, employment, individual income, health insurance, disclosure status.

Age was divided into two categories: <35 years and ≥35 years. Duration from HIV diagnosis and from ART initiation were divided into four categories: below 1 year, 1–2 years, 3–4 years and above 5 years; marital status was divided into three- married, unmarried and without a partner, unmarried with a partner, others (divorced, widowed, etc); Educational attainment of household members and individual income was divided into threelow, middle and high; Employment was divided into three- employed, unemployed and retired; finally, health insurance and disclosure status were divided into yes and no.

### Statistical analysis for the depression manuscript

Descriptive statistics, such as, mean, standard deviation (SD) for continuous variables, and frequency and proportion for categorical variables, were calculated among all subjects and by groups. For the BDI-II score, we performed explorative analysis using two-sample t-test, analysis of variance (ANOVA) or Pearson correlation. For CDRS-R score, we defined depression by having a score equal to or higher than 55. Chi-square test or two-sample t-test was used to explore the association between potential factors and depression.

The sociodemographic factors were age, sex, educational status, and marital status.

### Data Collection

Data were collected by four trained research assistants/data collectors (general nurses) using the Amharic version of the questionnaire for a month. The questionnaire was designed in English. The training was on introduction to depression and HIV comorbidity, research methods, interviewing skills, sampling and recruitment, and ethical aspects of research.

### Data Processing and Analysis

All collected data were checked for completeness and consistency and entered in to EPI INFO version 7 and then exported to SPSS for windows version 20 for analysis. Descriptive and bivariate logistic regression analyses were computed to see frequency distribution and to test whether there was an association between the independent and dependent variables, respectively. Factors associated with depressive symptoms were selected during bivariate analysis with a value of p≤0.2 for further analysis in multivariable regression analysis. Variables with P-value less than 0.05 at 95% confidence interval were considered as statistically significant.

### Ethical Approval

Ethical clearance was obtained from University of Nigeria Nsukka institutional review board. Permission was obtained from University of Nigeria, Nsukka Ethical Committee. Written consent was taken from study participants and assent from legally approved parents after explaining purpose of the study. Confidentiality was maintained by omitting their personal identification.

### Statistical methods

All survey data were cleaned and analyses were performed using SAS software v9.4. Depression was the outcome of interest and was calculated as the duration from the date of baseline interview until the date of depressive symptoms, if known. If only year of depressive symptoms was known, then the day and month were assigned at random with equal probability on all days of the year, or all days between the interview date and June 30, 2019 if the year of depressive symptoms and year of interview coincided.

Cumulative hazards were estimated using Nelson-Aalen cumulative hazards functions and hazard ratios were estimated using Cox proportional hazards models, with the social relationship measures, health and demographic covariates. The analysis was stratified by sampling strata defined by the impact of HIV/AIDS within each participant's family, allowing different baseline hazards for each stratum.

As is common in correlational study, we were unable to locate and follow up on a portion of the original cohort. The main analysis presented in the paper is of the participants whose survival status was known at follow-up. However, to assess the robustness of our findings from this complete-case analysis we also conducted a sensitivity analysis using multiple imputation to predict the vital status and observation time of the participants who were lost to follow-up. The imputation models for both vital status and observation time included sex, age, grip strength, hypertensive status, and all the social relationship variables.

## Results

### Participants for the Study

This study was conducted from January to June, 2019 on 1242 out-patients living with HIV/AIDS in three teaching hospitals in South Eastern Nigeria.

Table 1 presents the sociodemographic categorization of PLWHA. In relation to the individual determinants, 57% were females whilst 43% were males, 55% were below the age group of 35 years and 44% were above 35 years. Duration from HIV diagnosis reveals 1% for below one year, 9.7% for 1–2 years, 20% for 3–4 years and 69% for 5 years and above. Duration for ART initiation shows that 11% for below one year, 16% for 1–2 years, 33% for 3–4 years and 39% for 5 years and above. Moreover, those that are unmarried and without a partner had a high percentage of 81%, the married had 5%, unmarried but with a partner was 7% and also 7% for divorced and widowed. There was no much disparity in the low, middle and high educational attainment of the household members which revealed 50%, 32% and 17% respectively.

Their employment status revealed 40%, 42% and 17% for employed, unemployed and retired respectively. Their level of income shows that those with low, middle and high income had 45%, 39% and 17% respectively. The positive responses to health insurance was 42% and negative was 58% whilst the positive disclosure status was 73% and 27% was negative.

The main sources of instrumental social support were relatives not living in the same household (67%), this is followed by spouses/partners (47%), relatives living in the same household (41%), friends offered 31%, the boss/coworker, neighbours and health professionals offered very minimal instrumental social support of 4%, 5% and 9% respectively. The main sources of emotional support were relatives not resident in the same household (56%), friends (55%), and spouses/partners (50%). There was a low percentage of emotional social support from health professionals, neighbors, bosses/co-workers as 17%, 6% and 4% respectively.

Note: Participants may have indicated one or more sources of support.

By spouse/partner, instrumental social support (ISS) significantly predicted depression of the PLHIV (β=-.793, p=.000), while emotional social support (ESS) had significant predictive power of (β=-.833, p=.000) on depression of PLHIV. Apparently, both instrumental and emotional social supports significantly predict the depression of PLHIV.

By relatives living in the same house, ISS had significant predictive power on depression of PLHIV (β= -.893, p=.000), whilst ESS significantly predicts depression of PLHIV at (β= -.878, p=.000). For relatives not living in the same house, ISS and ESS significantly predicted depression of PLHIV (β=-.805, p=.000), and (β=-.793, p=.000), respectively

For friends with lower social support, the ISS predicted (β=-.476, p=.000), on depression of PLHIV with ESS showing (β=-.461, p=.000), for the co-workers, ISS significantly predicted depression (β=-.432, p=.000), while ESS predicted (β=-.489, p=.000) of PLHIV.

Neighbours predicted for ISS on depression of PLHIV (β=-.575, p=.000), while ESS predicted for health professionals (β=-.429, p=.000), ISS showed (β=-.465, p=.000) while ESS predicted the depression of PLHIV (β=-.489, p=.000).

The multiple regression of instrumental and emotional social supports predicting depression of PLHIV depicts the regression estimates and the adjusted R-square which reveals that 63%, 79%, 65%, 58%, 69%, 67% and 66% of prediction on depression were caused by poor instrumental social support respectively from the following sources: spouse/partner, relative living in the same house, relative not living in the same house, friends, boss/co-workers and neighbours. The emotional support reveals 69%, 77%, 63%, 57%, 68%, 53 and 56% predictive power on depression of PLHIV.

## Discussion of the findings

The findings of this study reveal our participants as more of females comprising 57% females and 43% males. The finding is in agreement with the report from National HIV and AIDS and reproductive health services of higher prevalence among women due to higher vulnerability and infections in all age groups^31^. One of the key drivers in HIV distribution is the entrenched danger of inequalities and inequities. This is mostly prevalent in the areas of economic dependency for women, because in most societies, men have greater control and access to productive resources 32. As a result of this, women do not have right to determine sex choice or right over their body. For instance, in sub-Saharan Africa, 61% of PLHIV are women^33^. Similarly, reports have shown that a young woman in Africa has up to eight times/span> likelihood to acquire HIV than a young man^34^. In Nigeria, HIV prevalence rate in females is 4.0% compared to 3.2% in men.

Duration of HIV diagnoses and duration for ART initiation revealed the greatest percentage for 5 years and above of 69% and 39% as against duration of 1 year to 5 years with 11%, 16%, 33%, whilst the duration for ART initiation showed 11% for below one year, 16% for 1–2 years, 33% for 3–4 years and 39%. This could be due to the time between the identification of HIV and the diagnoses which might take some time before the administration of medication. This aligns with 35 which reveals that the diagnosis of HIV includes testing services in health-care facilities, free-standing sites and a wide range of community-based approaches, as well as HIV self-testing which are done before announcing the HIV status of the individual (HIVST)^36^.

Moreover, those that are unmarried and without a partner had a high percentage of 81%, the married had 5%, unmarried but with a partner was 7% and also 7% for divorced and widowed. The high prevalence of HIV disease among the participants who are unmarried and without a partner could be attributed to their likelihood of having multiple partners than those married and with partners. This aligns with the studies which showed that in high burden areas of sub-Saharan Africa, younger individuals of 15–35 years of age are more likely to engage in risky sexual behavior, including multiple partners^37^ and carry the highest risk of becoming newly infected with HIV^38^. Younger men are also less likely to be aware of their HIV status^39^, and those infected with HIV are less likely to be on antiretroviral therapy (ART)40 and more likely to have detectable viral load.

The educational attainment of the level of education of the members of the household showed 50%, 32% and 17% for low, moderate and high respectively. The finding agrees with the study which reveals that level of educational attainment in the early 1990s, evidence suggested that populations with higher education levels were likely to have higher HIV rates. More recent evidence in countries such as Zambia and Uganda suggests that now, more years of education are increasingly associated with safer sexual behaviour and lower HIV prevalence. This is particularly true for young women with secondary education, who demonstrated significantly lower HIV prevalence rates than their peers who had dropped out of school earlier^41^.

The findings revealed the employment status as 40%, 42% and 17% for employed, unemployed and retired respectively. Their level of income shows that those with low, middle and high income had 45%, 39% and 17% respectively. Those with low income level seems to be more predisposed to HIV disease as they may be vulnerable to having sex with anyone who can offer money, not minding the HIV status. This aligns with the^42^ report which explained that “widespread poverty and unequal distribution of income that typify underdevelopment appear to stimulate the spread of HIV”^42^. Studies also show that in many African countries, the prevalence of HIV infection correlates directly with level of income. A study illustrated a strong positive relationship between household wealth and HIV infection prevalence in the United Republic of Tanzania^43^.

Another study also showed that national HIV prevalence rates appeared to correlate directly with national income across sub-Saharan Africa^44^. The positive responses to health insurance was 42%, while 58% was negative, the positive disclosure status was 73% whilst 27% was negative. This is in accordance with UNAIDS which reveals that an estimated 36.7 million people were living with HIV (PLHIV) globally at the end of 2015, whilst 17 million PLHIV were accessing antiretroviral therapy (ART). It was only 46 percent of all PLHIV who needed the ART were able to access ART. It was shown that even when ART is provided for free by governments, often in partnership with donors/international organizations, PLHIV need affordable medical insurance to cover other essential costs, including the cost for treatment of opportunistic infections and out of pocket expenses related to treatment. HIV-related illnesses continue to be disproportionately excluded from public and private sector insurance policies and schemes. Research has revealed that PLHIV are often denied insurance coverage because of their pre-existing condition or are covered at a higher cost. Insurers perceive covering PLHIV to be a risky business and costly for them^45^.

The main sources of instrumental social support were relatives not living in the same household, followed by spouse/partners, relatives living in the same household, and friends. For the emotional social support, the major source of the social support were relatives not living in the same household, friends, spouse/partner and relatives living in the same household. There was lack of instrumental and emotional social supports from boss/co-workers, neighbours and health professionals.

The reason for the relatives not living in the same household, spouses/partners, relatives living in the same household, and friends being the most mentioned people, could be attributed to AIDS which might be occurring amongst couples with different HIV status, where there is a tendency of not revealing the diagnosis due to stigma and fear of abandonment^46^. Another reason could be that infection was contracted by the partner him/herself, which can weaken the bond and hinder the availability of social support available.

PLHIV may have fear of rejection, fear of co-workers, relatives and other people turning away from them and the fear of being victims of prejudice in society, which could make them choose rather not to reveal the HIV positive serology diagnosis and to seek ways to hide the diagnosis from other people^47^. That fear may be the reason why neighbors and employers/coworkers were less mentioned as a source of social support. The finding of this study indicates that the boss/co-workers, neighbours, and health professionals were less mentioned, both from instrumental and emotional social support, indicating sources of lack of social support. In the working environments, weaknesses were reported with specific issues related to HIV, such as the fear of disease transmission at the workplace^48^. Contradictorily, research developed with teachers that were evaluating the social support received at work, showed high appreciation of emotional support^49^. This variation could be because the study was conducted in an environment that did not involve people with HIV. The relationship with the health professional and the support expected by the patient interferes with treatment compliance. By ensuring that an effective doctor-patient relationship affects the evolution of the disease is to fully endorse that, which is associated with better health^49^.

The spouse/partner's instrumental and emotional social supports significantly predicted depression of PLHIV. Also the relatives living in the same house and relatives not living in the same house, friends, boss/co-workers, neighbours and health professionals significantly predicted depression of PLHIV. This finding seems to be in agreement with previous findings, which suggested social support, especially emotional support, buffers the deleterious influences of stressful events and reduces the risk of depression. The finding reveals the family to be the vital source of all types of social support among HIV patients; it was found that participants who lacked tangible support, affectionate support, and positive social interaction from their family had a higher likelihood of having depression. On the other hand, another study found that women with a closer connection to their spouses reported lower depressive symptoms, while the relationship between spousal support and men's depressive symptoms was more complex, being affected by their perceived independence (i.e., having no need for emotional support) and by the nature of their attachment to their wives^50^. Unarguably, communication with families is a key means to reduce the incidence of depression of patients. Although it has been well-known that forms of family-based intervention, such as involvement of family in HIV therapy or provision of educational programs for family, could help foster patients' mental health^51^;^52^;^53^, people outside the family may also be important predictors. Previous studies showed that PLHIV have been severely discriminated against not only by the general population^54^, but also by healthcare providers^55^. This study found potential effectiveness of social support as an alleviator of depression, and recognized the importance of people outside the family as a source of social support. The findings of our study could provide relevant information for future mental health strategies in other resource-constrained settings and in family-oriented societies.

Finally, we did not assess supporters' relationships and dynamics in relation to depression. Such information is relevant for formulating a strategic plan to address depression. Conclusively, analyses identified knowledge gaps in the community regarding the social support received by PLHIV and their depression symptoms. We recommend that both instrumental and emotional social supports should be offered to the PLHIV by their family members, the health professionals, boss/coworkers, friends and everybody around them so as to give them psychological relief and save them from depression.

## Figures and Tables

**Table 2:** Sources of instrumental and emotional social supports of people living with HIV/AIDS

Source of Social support		Social support

	Instrumental n (%)	Emotional n (%)
Spouse/partner	53 (47)	57 (50)
Relatives living in the same household	46 (41)	44 (39)
Relatives not living in the same household	76 (67)	64 (56)
Friend(s)	35(31)	63 (55)
Boss/co-worker	4 (4)	4 (4)
Neighbor(s)	5 (5)	5 (6)
Health professionals	10 (9)	19 (17)

**Table 3:** Hierarchical Regression of the predictive factors of instrumental and emotional social supports on depression of people living with HIV

	*Instrumental S/Support*				*Emotional S/Support*			
	
Variable	R^2^	Adj. R^2^	β	*p*	R^2^	Adj. R^2^	β	*p*
**Spouse/Partner**	.628	.627	-.793	.000	.694	.693	-.833	.000
**Relative living in** **the same house**	.791	.789	-.893	.000	.771	.768	-.878	.000
**Relatives not** **living in the same** **House**	.648	.647	-.805	.000	.629	.628	-.793	.000
**Friends**	.480	.576	-.476	.000	.578	.566	-.461	.000
**Boss/co-workers**	.474	.685	-.432	.000	.488	.681	-.489	.000
**Neighbours**	.476	.665	-.575	.000	.406	.531	-.429	.000
**Health** **Professionals**	.474	.660	-.465	.000	.452	.561	-.489	.000
